# Effects of agronomic traits and climatic factors on yield and yield stability of summer maize (*Zea mays* L) in the Huang-Huai-Hai Plain in China

**DOI:** 10.3389/fpls.2022.1050064

**Published:** 2022-11-10

**Authors:** Hao Ren, Mingyu Liu, Jibo Zhang, Peng Liu, Cunhui Liu

**Affiliations:** ^1^ College of Agronomy, State Key Laboratory of Crop Biology, Shandong Agricultural University, Tai’an, Shandong, China; ^2^ Shandong Climate Center, Jinan, Shandong, China; ^3^ Shandong Seed Administration Station, Shandong Provincial Department of Agriculture and Rural Affairs, Jinan, Shandong, China

**Keywords:** agronomic traits, climatic factors, yield, yield stability, summer maize

## Abstract

Zhengdan 958 (ZD958) is the summer maize variety with the widest planting area in Huang-Huai-Hai plain in the past 20 years. Understanding the agronomic characteristics of maize and its adaptability to climatic factors is of great significance for breeding maize varieties with high yield and stability. In this study, the experimental data of 33 experimental stations from 2005 to 2015 were analyzed to clarify the effects of different agronomic traits on yield and the correlation between agronomic traits, and to understand the effects of different climatic factors on summer maize yield and agronomic traits. The results showed that the average yield of ZD958 was 9.20 t ha^-1^, and the yield variation coefficient was 13.41%. There was a certainly negative correlation between high yield and high stability. Plant heights, ear heights, double ear rate, ear length, ear rows, line grain number, grain number per ear, ear diameter, cob diameter, and 1000 grains weight were significantly positive correlation with maize yield. Solar radiation before and after silking were significantly positive correlation with maize yield. Path analysis showed that changes in agronomic traits accounted for 54% of the yield variation, and changes in climate factors accounted for 26% of the yield variation. Our study showed that higher plant height, ear height, grain number per ear and 1000-grain weight, lower lodging rate, pour the discount rate and shorter bald tip long were the main reasons for high yield. Among the climatic factors, solar radiation and the lowest temperature have significant effects on the yield.

## Introduction

With the rapidly population growth, the demand for food will continue to increase in the future. In China, increasing maize (*Zea mays* L) yield per unit area is the only way to increase production under the condition of decreasing planting area (Chen et al., 2021). Crop improvement has long created basic wealth that supports social development, human well-being, and adaptation to environmental change. Improved agronomic traits of maize have taken place for thousands of years (Simmons et al., 2021). The contribution of breeding to maize yield is 51% in China (Liu et al., 2021b), while 50% in the United States (Duvick, 2005; Lee and Tollenaar, 2007). Therefore, the improvement of agronomic traits is still an important measure to increase maize yield.

The term ‘Agronomc Traits’ has varied definitions. Agronomic traits include plant structure, maturation traits, physiological traits that drive intrinsic performance of plants, and even molecular or physiological mechanisms, which involve all aspects of plant biology (Simmons et al., 2021). The selection of traits needs to be easy to measure and identify, closely related to desired parameters, and has a high heritability, allowing breeders to make greater progress in the shortest possible time (Setimela et al., 2017). Understanding the molecular mechanisms of important agronomic traits is significant for breed selection. Wang et al. (2019) reported that spatiotemporal expression of genes involved in a variety of plant hormone pathways is an important pathway affecting plant height. Li et al. (2019) suggested ZMRPH1 plays a vital role in maize polar cell growth control and encodes a microtubule-associated protein that controls plant height and ear height. Jia et al. (2020) reported that KNR6 can interact with Arf GTPase- activating protein, and its phosphorylation may affect ear length and grain numbers. These findings provide theoretical basis for increasing grain yield of maize hybrids.

Before analyzing the molecular mechanisms of agronomic traits, it is important to study the relationships between traits through correlation coefficients for early selection of plants or inbred lines, or simultaneous selection of multiple traits (Falcon et al., 2020). Path analysis can prove the positive and negative correlations, high and low intensities between traits under study, it is an important tool to help breeders determine the preferred traits (Silva et al., 2016). ZD958, which has been widely used in Huang-Huai-Hai region for many years (Ren et al., 2021), which was used as the experimental material and conducted a series of multi-point variety experiments in recent years. The main purpose of this study is (1) to analyze the yield and stability of ZD958 in Huang-Huai-Hai region; (2) to clarify the relationships between different agronomic traits; (3) to understand the effects of climate factors on the yield and agronomic traits of summer maize. This study provides theoretical support for breeding cultivars with high stability and high climate suitability.

## Materials and methods

### Experimental region

The experimental sites were in Shandong Province, China, which is one of the main provinces in the Huang-Huai-Hai Plain. The Huang-Huai-Hai Plain is the main maize planting region in China. Which is a temperate monsoon climate zone with high temperature and rainy summer. The detailed climatic conditions are shown in **Table S1**.

### Experimental design

**Figure 1:**
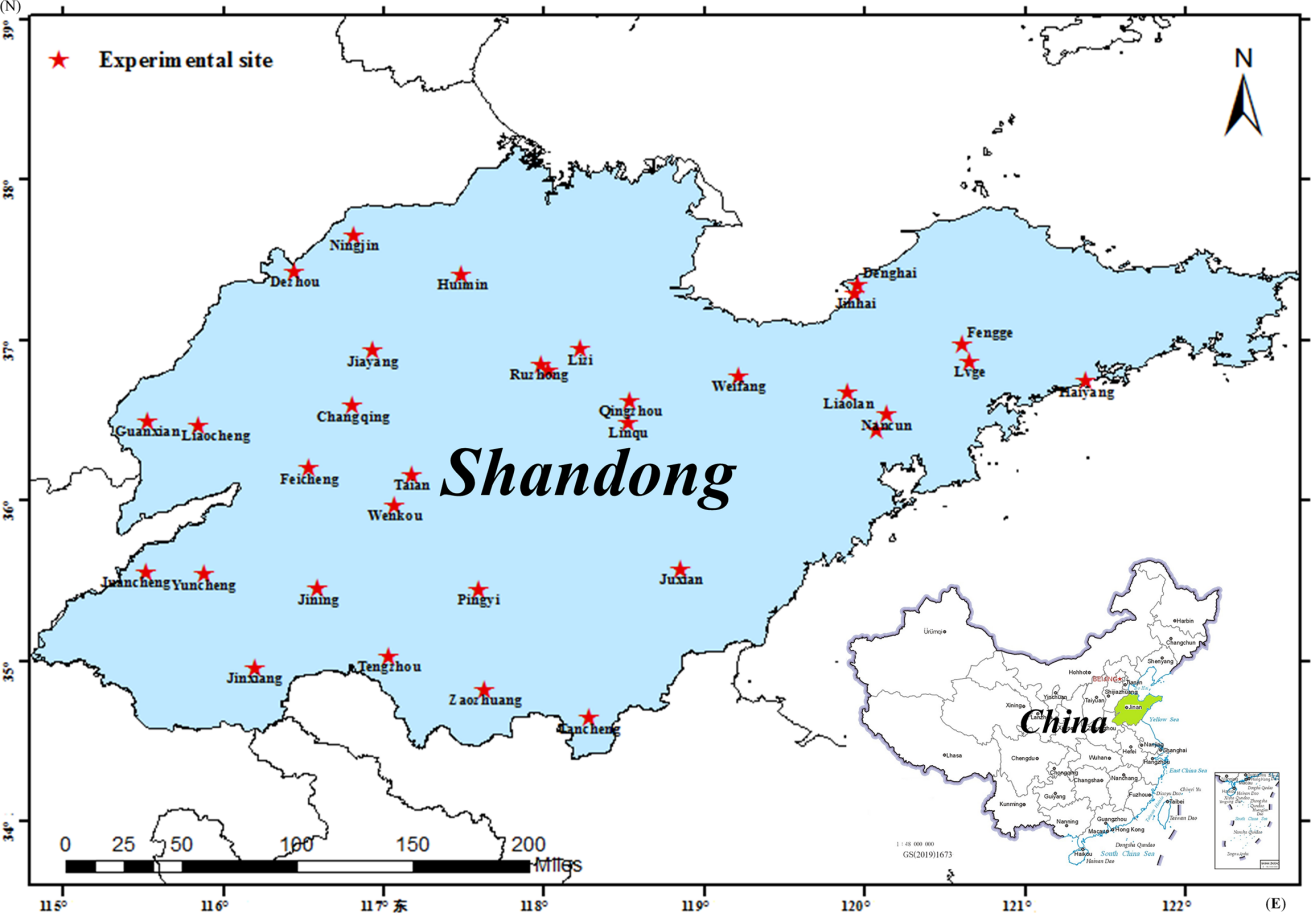
Distribution of experiment sites. The red star represents the experiment site.

ZD958 was used in the experiment, which is a maize cultivar planted in a large area in Huang-Huai-Hai region. The planting density was 67,500 plants ha^-1^. The distribution of the experimental sites is shown in **Figure 1**. Yield experiments were conducted at the above test stations from 2005 to 2015, respectively. At least five replicates were set in each experimental site, and the area of each cell was no less than 300 m^2^. Before sowing, 315 kg N ha^-1^, 180 kg P ha^−1^,180 kg K ha^−1^ were applied as urea (46% N), calcium superphosphate (12% P_2_O_5_), and potassium sulfate (51% K_2_O) respectively. Flood irrigation was selected as the irrigation method, and supplementary irrigation was carried out in time according to the demand of maize growth and climatic conditions.

### Phenological development and calculation of climatic factors

Days from sowing to silking (DTS) was recorded when 50% of the ear in each plot had stigma filiform protruding from tip of bract in bundles, while days from sowing to maturity (DTM) was defined to be the date when the milkline on the grain disappears and the black layer appears (Carter and Poneleit, 1973). The duration of flowering to maturity was calculated by the difference between DTM and DTS. The climatic factors are divided into different stages for analysis. We collected daily weather data of each experimental station from the Shandong Meteorological Center in China. The datum includes average temperature, maximum and minimum temperature, rainfall, and solar radiation (**Table S1**). These meteorological factors were decomposed into rainfall after silking (RainA), rainfall before silking (RainB), rainfall in total growth stage (RainT), solar after silking (SolarA), solar before silking (SolarB), solar in total growth stage (SolarT), effective accumulative temperature after silking (TeffectA), effective accumulative temperature before silking (TeffectB), effective accumulative temperature in total growth stage (TeffectT), Maximum accumulated temperature after silking (TmaxA), Maximum accumulated temperature before silking (TmaxB), Maximum accumulated temperature in total growth stage (TmaxT), Minimum cumulative temperature after silking (TminA), minimum cumulative temperature before silking (TminB), minimum cumulative temperature in total growth stage (TminT) according to maize silking stage.

### Plant sampling

At the end of the growing season, all plants in three rows of 5 m (Three repeats) in the center of each plot were harvested at ground level to minimize edge effects. The ear numbers were counted in a 9 m^2^ subplot of each plot and 10–15 of the harvested plants was randomly selected and their plant heights (PH), ear heights (EH), stem diameter (SD) were measured. Before harvest, double ear rate (DER), empty ear rate (EER), lodging rate (LR), pour the discount rate (PDR) and maize rough dwarf virus (MRDV) in the plot were investigated. Ear length (EL), ear rows (ER), line grain number (LGN), grain number per ear (GNE), ear diameter (ED), cob diameter (CD), bald tip long (BTL), kernel percentage (KP), 1000 grains weight (GW1000) and grain bulk density (GBD) were measured manually after harvest. In this study, the water content of grain yield was calculated as 14%.

### Statistical analysis

In this study, yield coefficient of variation (CV) and yield sustainability index (SYI) were used to represent the yield stability state of maize, as shown in the period calculation method below:

CV = Yield standard deviation/Mean yield × 100%
SYI = (Mean yield - Yield standard deviation)/Max yield × 100%

Software DPS 17.10 was used for ANOVA and path (stepwise regression) analysis. The correlation analysis was carried out using the Pearson correlation method with the Hmisc package of R for 3.6.1. Plot using R for 3.6.1.

## Results

### Grain yields and yield stability

**Figure 2:**
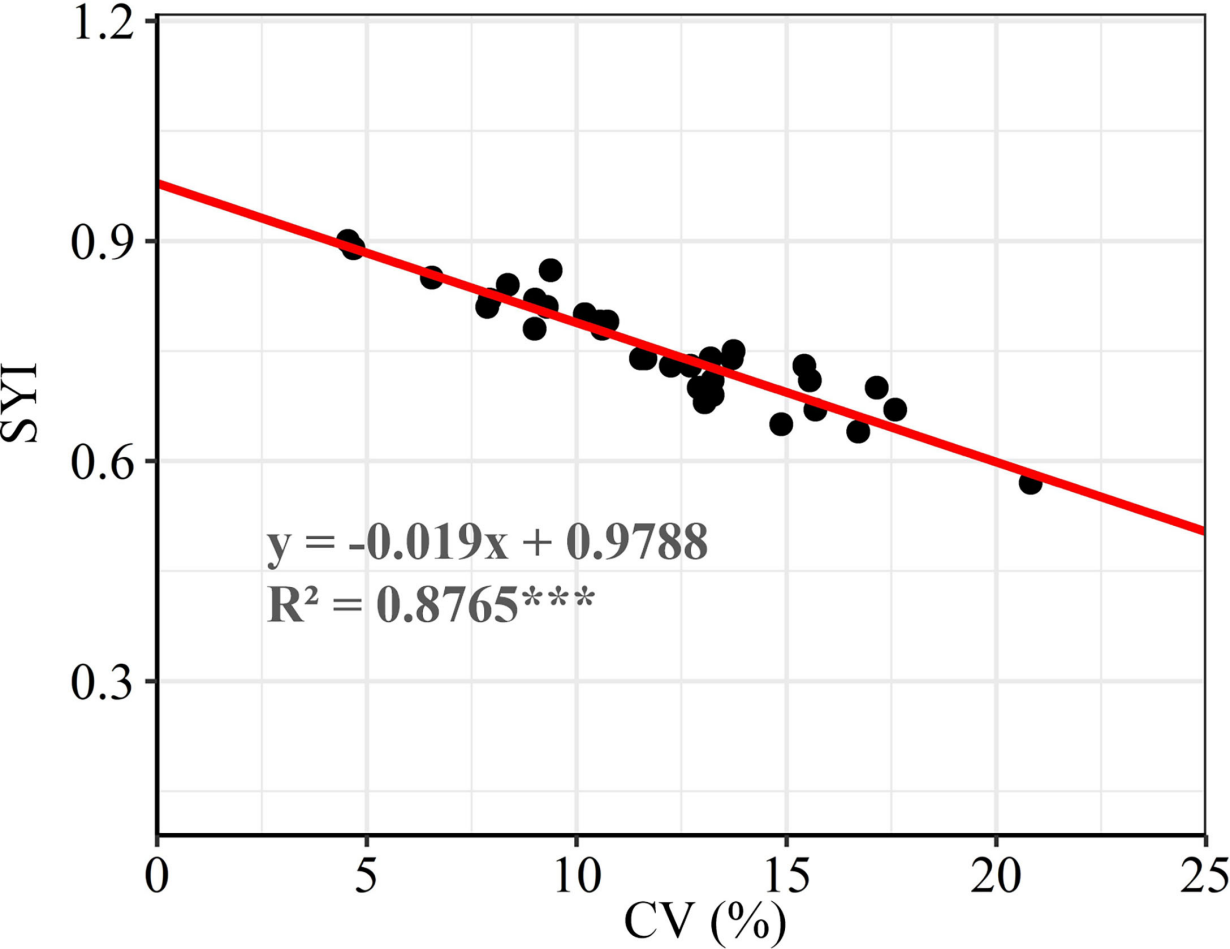
Correlation analysis between SYI and CV. *** means significant at the *P<0.001* level.

**Figure 3:**
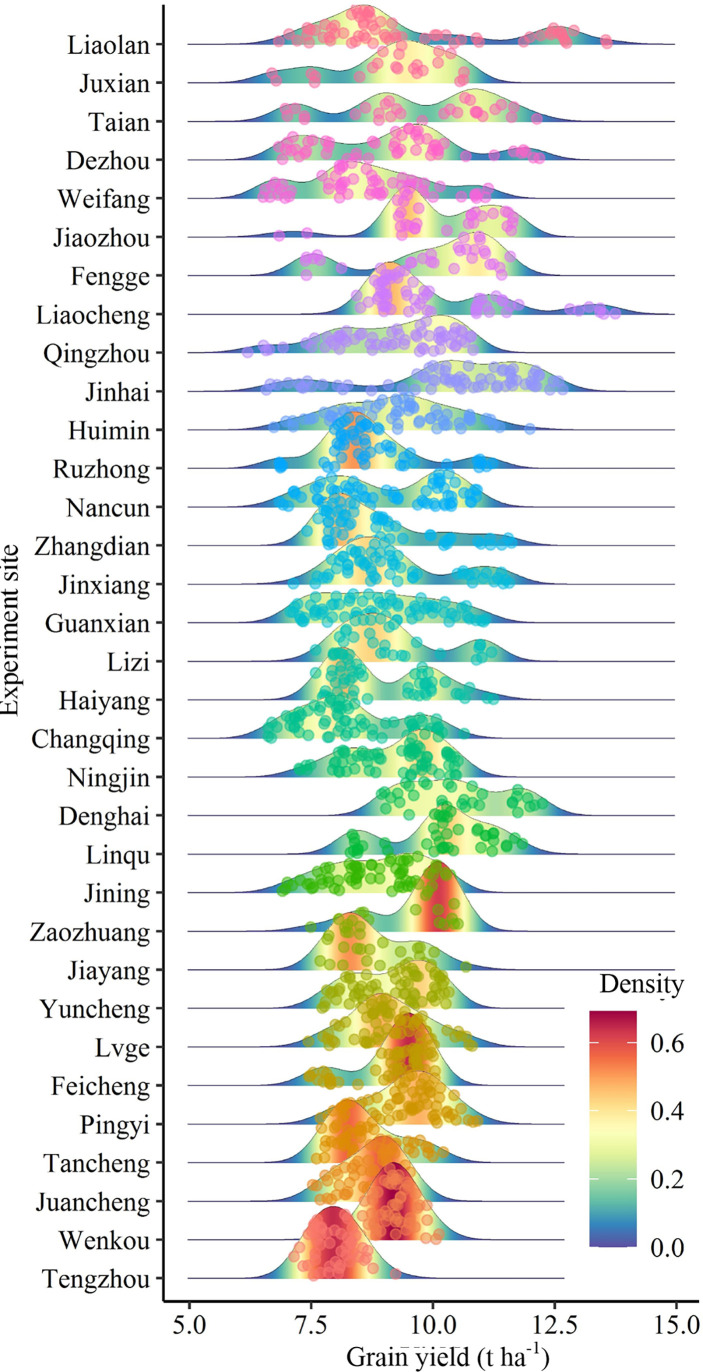
Analysis of yield distribution at different experiment sites. The redder the color and the higher the peak, the more points of distribution.

There were significant differences in the grain yield of summer maize among different experimental sites. The summer maize yield in Denghai and Jinhua was significantly higher than that in other regions. The yield gaps between regions was about 2.48 t ha^-1^. According to the ranking of regions by CV, it shows that the CV and SYI of yield between regions were greatly different. There was a significant negative correlation between CV and SYI (**Figure 2**). CV was lower, and SYI was higher in Tengzhou, while that of Liaolan was contrary (**Table 1**). Areas with large yield CV are more likely to have high yield, while areas with small yield CV have low yield but strong stability (**Figure 3**).

**Table 1:** Yield and yield stability of maize in different experimental sites.

Site	Mean yield (kg ha^-1^)		Max yield (kg ha^-1^)	Min yield (kg ha^-1^)	Standard deviation (kg ha^-1^)	CV (%)	SYI
Tengzhou	8.00	e	8.51	7.65	0.36	4.55	0.9
Wenkou	9.13	bcde	9.82	8.38	0.43	4.68	0.89
Juancheng	8.72	de	9.55	7.75	0.57	6.55	0.85
Tancheng	8.63	de	9.84	7.85	0.68	7.87	0.81
Pingyi	9.42	abcd	10.53	8.10	0.75	7.93	0.82
Feicheng	9.18	bcd	9.97	7.60	0.77	8.36	0.84
Lvge	9.06	cde	10.58	7.80	0.82	9.00	0.78
Yuncheng	9.21	bcd	10.16	7.87	0.83	9.01	0.82
Jiayang	8.82	de	9.84	7.88	0.82	9.29	0.81
Zaozhuang	9.67	abcd	10.20	8.32	0.91	9.38	0.86
Jining	9.02	cde	10.16	7.35	0.92	10.2	0.8
Linqu	10.06	abc	11.39	8.39	1.06	10.56	0.79
Denghai	10.48	a	12.01	9.08	1.11	10.61	0.78
Ningjin	9.20	bcd	10.36	7.36	0.99	10.74	0.79
Changqing	8.15	e	9.78	6.92	0.94	11.53	0.74
Haiyang	8.97	cde	10.72	7.80	1.04	11.64	0.74
Lizi	9.08	bcde	10.87	8.08	1.11	12.25	0.73
Guanxian	8.88	de	10.64	7.27	1.13	12.71	0.73
Jinxiang	9.13	bcde	11.37	7.81	1.18	12.91	0.70
Zhangdian	8.86	de	11.26	7.84	1.16	13.05	0.68
Nancun	9.14	bcde	10.80	7.20	1.21	13.19	0.74
Ruzhong	8.63	de	10.91	6.87	1.14	13.24	0.69
Huimin	9.11	bcde	11.17	7.16	1.21	13.24	0.71
Jinhai	10.43	a	12.14	7.28	1.43	13.70	0.74
Qingzhou	9.10	bcde	10.42	6.57	1.25	13.74	0.75
Liaocheng	10.11	ab	13.3	8.82	1.50	14.88	0.65
Fengge	9.71	abcd	11.24	7.47	1.50	15.43	0.73
Jiaozhou	9.63	abcd	11.52	7.13	1.50	15.56	0.71
Weifang	8.63	de	10.90	6.73	1.35	15.69	0.67
Dezhou	9.11	bcde	11.81	7.13	1.52	16.71	0.64
Taian	9.48	abcd	11.20	7.17	1.63	17.15	0.70
Juxian	9.64	abcd	11.92	7.22	1.70	17.59	0.67
Liaolan	9.35	abcd	12.89	7.12	1.95	20.82	0.57

Symbols not sharing any lowercase letters are significantly different at the level of p < 0.05.

### Relationship between maize plant agronomic traits and yield

**Figure 4:**
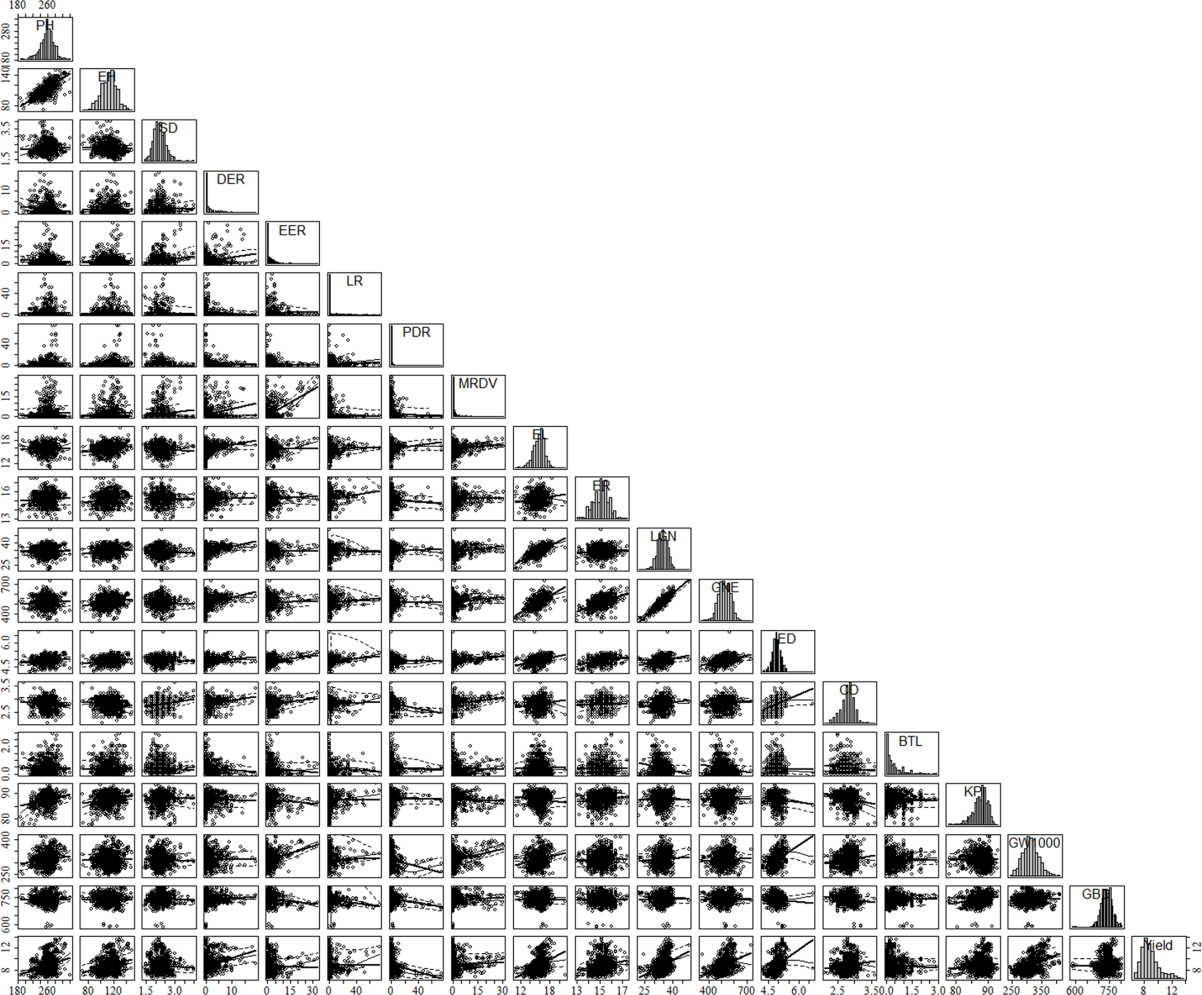
Scatterplot matrix of plant agronomic traits and yield based on literature data. The matrix was created by using the car package in R software. Diagonal panels show the distribution of each variable. Lower panels show the scatterplot between two different parameters: x-axis, column variable; y-axis, row variable. PH, plant height, cm; Ell, ear height, cm; SD, stem diameter, em; DER, Double ear rate, %; EER, empty ear rate, %; IR, lodging rate, %; PDR. Pour the discount rate, %; MRDV, maize rough dwarf virus, %; EI, var length, cm; ER, car rows; LGN. line grain number; GNE, grain number per ear; ED, ear diameter, cm; CD, cob diameter, cm; BTL, bald tip long, cm; KP, kernel percentage, %; GW1000, 1000 grains weight, g: GBD, grain bulk density, g L^-1^.

The agronomic traits of maize were closely related to their yield. In this study, the main agronomic characters of other maize were analyzed statistically based on multi-point experimental data. The results show that maize yield was significant positively correlated with PH, EH, DER, EL, ER, LGN, GNE, ED, CD, GW1000, and negatively correlated with SD, EER, PDR, BTL (**Figure 4**). There were also significant correlations between agronomic traits, such as PH was positively correlated with EH, PDR, MRDV, EL, ER, LGN, GNE, KP, and GW1000, and significantly negatively correlated with GBD (**Table 2**). The agronomic traits of maize in different environments had great variation. The DER, EER, LR, PDR and MRDV distribution of ZD958 tended to 0, while other traits showed normal distribution (**Figure 4**). The coefficients of variation of PH, EH, SD, DER, EER, LR, PDR, MRDV, EL, ER, LGN, GNE, ED, CD, BTL, KP, GW1000 and GBD were 6.37%, 11.13%, 15.66%, 168.06%, 192.41%, 306.03%, 265.42%, 285.10%, 7.08%, 4.63%, 8.26%, 9.84%, 4.59%, 6.82%, 108.09%, 2.64%, 8.55%, 3.95%, 14.18%, respectively. The mean values of PH, EH, SD, DER, EER, LR, PDR, MRDV, EL, ER, LGN, GNE, ED, CD, BTL, KP, GW1000 and GBD were 258.05 cm, 111.46 cm, 2.28 cm, 1.48%, 1.55%, 2.22%, 2.29%, 1.74%, 16.17 cm, 15.14, 35.03, 529.60, 4.87 cm, 2.79 cm, 0.42 cm, 87.69%, 316.69 g, 741.80 g L^-1^, respectively.

**Table 2:** Matrix of correlation coeffcients between plant agronomic traits and yield of maize based on linear relationships.

	PH	EH	SD	DER	EER	LR	PDR	MRDV	EL	ER	LGN	GNE	ED	CD	BTL	KP	GW1000	GBD	Yield
PH	1.00																		
EH	0.69^***^	1.00																	
SD	-0.03	-0.10^***^	1.00																
DER	0.06	0.07^*^	-0.02	1.00															
EER	-0.07	0.03	0.22^***^	0.18^***^	1.00														
LR	0.07	0.06	0.02	-0.17^**^	0.00	1.00													
PDR	0.12^***^	0.11^***^	-0.08	0.07	-0.07	0.14^**^	1.00												
MRDV	0.11^**^	0.08^*^	0.01	0.19^***^	0.43^***^	0.01	0.01	1.00											
EL	0.12^***^	0.10^***^	-0.04	0.20^***^	0.00	0.04	0.09^*^	0.08	1.00										
ER	0.15^***^	0.18^***^	-0.03	0.06	0.06	0.16^***^	-0.08^*^	0.00	0.16^***^	1.00									
LGN	0.12^***^	0.07^**^	-0.04	0.25^***^	-0.05	-0.01	0.05	0.09	0.64^***^	0.12^***^	1.00								
GNE	0.15^***^	0.17^***^	-0.05	0.20^***^	-0.01	0.06	0.02	0.09	0.58^***^	0.55^***^	0.86^***^	1.00							
ED	0.20^***^	0.26^***^	-0.07^*^	0.15^***^	0.23^***^	0.12^**^	-0.02	0.18	0.29^***^	0.33^***^	0.18^***^	0.29^***^	1.00						
CD	-0.04	0.01	0.14^***^	0.14^***^	0.15^***^	0.02	-0.11^**^	0.12	0.09	0.09^***^	0.04	0.08^**^	0.38^***^	1.00					
BTL	-0.02	-0.02	-0.02	-0.10^**^	-0.04	0.02	-0.05	0.02	-0.11	-0.01	-0.33^***^	-0.28^***^	-0.12^***^	-0.10^***^	1.00				
KP	0.11^***^	0.09^***^	0.05	-0.08^*^	-0.22^***^	0.06	0.01	-0.09	-0.09	0.03	0.01	0.02	-0.12^***^	-0.18^***^	-0.07^*^	1.00			
GW 1000	0.09^***^	0.03	-0.04	0.05	0.27^***^	0.05	-0.21^***^	0.15	0.26^***^	0.05^*^	0.13^***^	0.11^***^	0.36^***^	0.12^***^	-0.08^**^	-0.07^*^	1.00		
GBD	-0.07^**^	-0.15^***^	0.02	0.03	-0.17^***^	-0.11^*^	-0.11^***^	-0.03	0.01	-0.08^***^	0.04	-0.03	-0.20^***^	-0.16^***^	0.02	0.28^***^	0.09^***^	1.00	
Yield	0.37^***^	0.26^***^	-0.12^***^	0.25^***^	-0.08^*^	0.03	-0.16^***^	0.02	0.44^***^	0.20^***^	0.45^***^	0.45^***^	0.34^***^	0.06^**^	-0.16^***^	0.04	0.43^***^	0.01	1.00

PH, plant height; EH, ear height; SD, stem diameter; DER, Double ear rate; EER, empty ear rate; LR, lodging rate; PDR, Pour the discount rate; MRDV, maize rough dwarf virus; EL, ear length; ER, ear rows; LGN, line grain number; GNE, grain number per ear; ED, ear diameter; CD, cob diameter; BTL, bald tip long; KP, kernel percentage; GW1000, 1000 grains weight; GBD, grain bulk density. *, significant correlation at the level of p < 0.05; **, significant correlation at the level of p < 0.01; ***, significant correlation at the level of p < 0.001.

**Figure 5:**
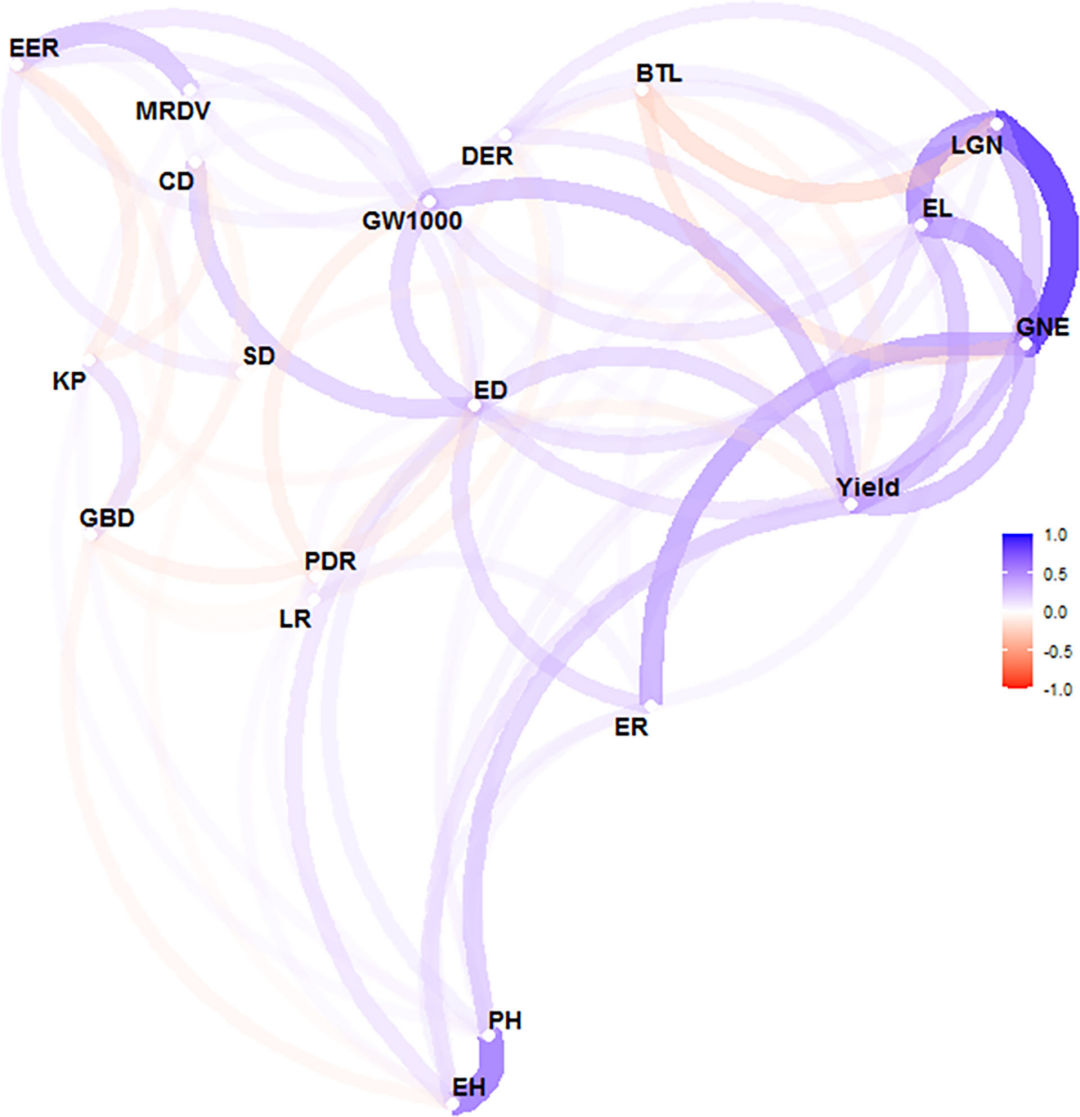
Correlation analysis of agronomic characters and yield of maize. The correlation was conducted by using the 'corrr' package in R software. The closer variables represent that these two variables are highly linearly correlated, while the opposite is the case for widely spaced variables. The line color represents the direction of the correlation. The blue line is positive correlation and the red line is negative correlation. The line shading and thickness represent the strength of the relationship. The minimum correlation coeffcient required to display a line between variables is 0.1. PH, plant height, cm; EH, ear height, cm; SD, stem diameter, cm; DER, Double ear rate, %; EER, empty ear rate, %; LR, lodging rate, %; PDR. Pour the discount rate, %; MRDV, maize rough dwarf virus, %; EL, ear length, cm; ER, ear rows; LGN, line grain number; GNE, grain number per ear: ED, ear diameter, cm; CD, cob diameter, cm; BIL, bald tip long, cm; KP, kernel percentage. %; GW1000, 1000 grains weight, g; GBD, grain bulk density, g L^-1^.

**Figure 6:**
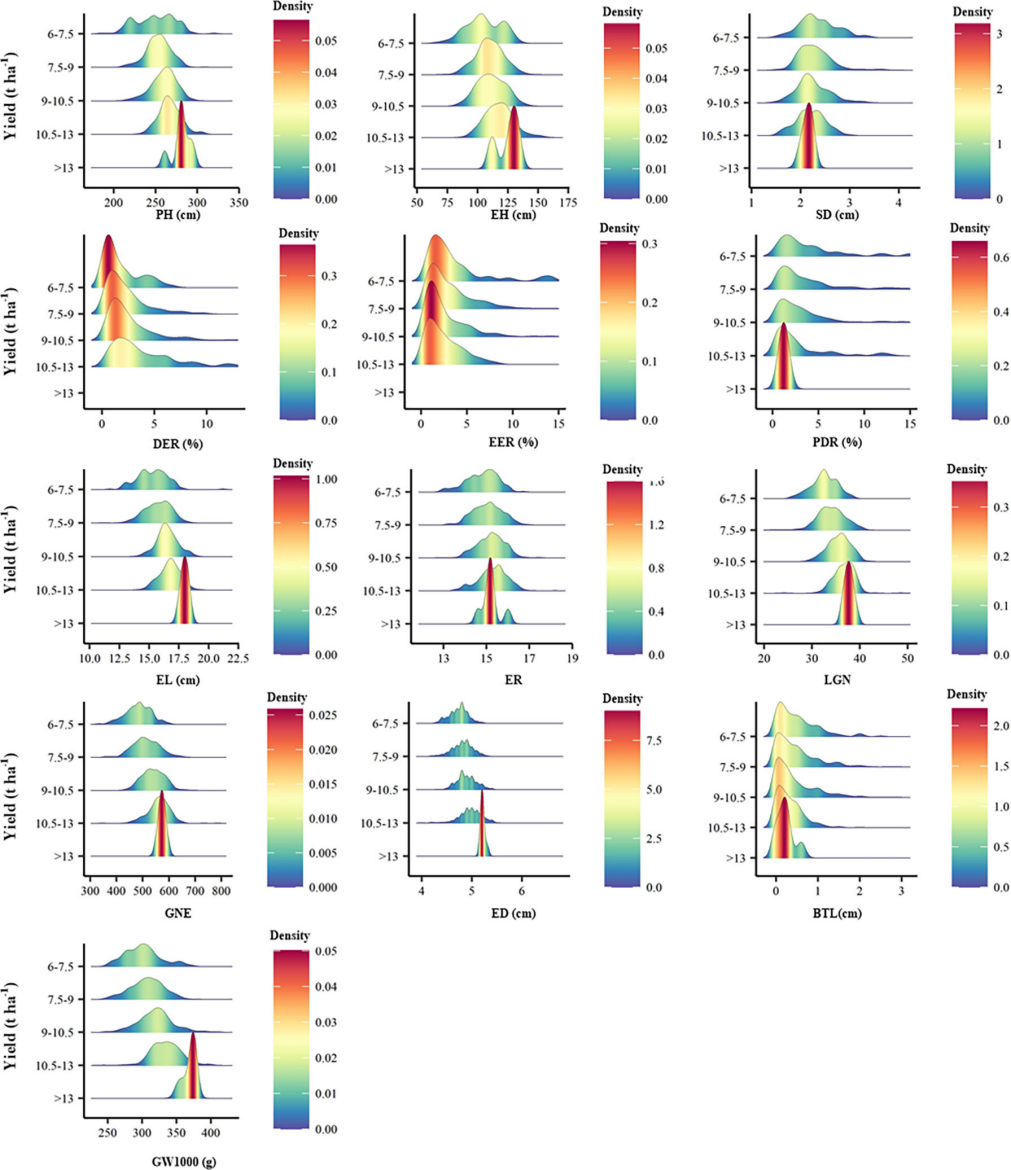
Peak map of distribution of agronomic traits of different yield levels. PH, plant height, cm; EH, ear height, cm; SD, stem diameter, cm; DER, Double ear rate, %; EER, empty ear rate, %; PDR, Pour the discount rate, %; EL, ear length, cm; ER, ear rows; LGN, Line grain number; GNE, grain number per ear; ED, ear diameter, cm; BTL, bald tip long, cm; GW1000, 1000 grains weight, g.

There is no significant correlation between yield and KP and GBD, and in addition, the long distance between yield and these two parameters indicates a large variability between them. GW1000, ED, EL, LGN and GNE were the main factors affecting the yield (**Figure 5**). When the yield was >13 t ha^-1^, the increase of ER and GW1000 was the main reason for the higher yield. The comparison of agronomic traits among different yield levels showed that there were significant differences among different yield levels for PH, EH, SD, DER, EER, PDR, EL, ER, LGN, GNE, ED, BTL and GW1000 (**Table 3**). There were also differences in the distribution centers of high nuclear density among the above agronomic traits with different yield levels (**Figure 6**).

**Table 3:** Agronomic traits of plants at different yield levels.

Yield (t ha^-1^)	6-7.5	7.5-9	9-10.5	10.5-13	>13	P-value
PH (cm)	249.08±23.62	253.59±15.14	260.20±14.75	266.82±13.22	281.14±10.65	< 0.001^***^
EH (cm)	105.38±14.70	109.98±11.64	111.36±11.78	117.70±11.79	124.71±9.09	< 0.001^***^
SD (cm)	2.36 ±0.38	2.30±0.36	2.25±0.35	2.20±0.29	2.14±0.09	0.005^**^
DER (%)	0.54±1.25	0.89±1.62	1.61±2.50	2.64±3.36	0.17±0.45	< 0.001^***^
EER (%)	2.31±3.40	1.56±2.55	1.29±3.18	1.36±2.20	0.00±0.00	< 0.001^***^
LR (%)	1.48±4.42	1.86±5.62	1.51±4.15	1.91±7.83	0.34 ±0.91	0.571
PDR (%)	4.46±11.65	2.14±5.42	1.56±3.67	0.96±2.74	0.51±0.64	< 0.001^***^
MRDV (%)	1.31±2.60	1.68±4.50	1.65±5.36	1.39±3.09	0.00±0.00	0.634
EL (cm)	15.27±1.31	15.72±1.17	16.51±0.88	16.74±0.88	17.97±0.30	< 0.001^***^
ER	14.80±0.74	15.06±0.70	15.20±0.67	15.31±0.68	15.20±0.42	< 0.001^***^
LGN	32.57±2.71	34.04±2.98	35.64±2.43	36.73±2.20	37.71±0.92	<0.001^***^
GNE	482.93±49.40	512.93±52.30	539.73±43.03	561.31±44.22	573.06±12.12	< 0.001^***^
ED (cm)	4.73±0.18	4.81±0.22	4.89±0.20	4.97±0.23	5.21±0.04	< 0.001^***^
CD (cm)	2.76±0.19	2.80±0.19	2.79±0.19	2.81±0.20	2.83±0.10	0.116
BTL (cm)	0.46±0.48	0.41±0.49	0.37±0.46	0.31±0.31	0.21±0.20	< 0.001^***^
KP (%)	87.49±2.84	87.68±2.36	87.67±2.20	87.82±2.20	88.97±0.66	0.348
GW1000 (g)	301.27±26.11	307.23±24.27	319.90±26.37	334.74±21.24	369.06±9.02	< 0.001^***^
GBD (g L^-1^)	743.20±23.68	739.68±29.49	743.77±31.73	740.66±25.60	760.46±16.00	0.051

PH, plant height; EH, ear height; SD, stem diameter; DER, Double ear rate; EER, empty ear rate; LR, lodging rate; PDR, Pour the discount rate; MRDV, maize rough dwarf virus; EL, ear length; ER, ear rows; LGN, line grain number; GNE, grain number per ear; ED, ear diameter; CD, cob diameter; BTL, bald tip long; KP, kernel percentage; GW1000, 1000 grains weight; GBD, grain bulk density. **, significant correlation at the level of p < 0.01; ***, significant correlation at the level of p < 0.001.

### Correlation between climatic factors and agronomic traits of maize plants

Changes in agronomic traits can be significantly affected by climatic factors. PH will be significantly positively correlated with RainA, RainB, RainT, TeffectA, TeffectB, TeffectT, TminB, TminT, and significantly negatively correlated with SolarB and SolarT. For the yield, RainA, RainB, RainT, TeffectB, and TeffectT had negative effects, while SolarA, SolarB, SolarT, and TeffectA had positive effects. The relationship between agronomic traits and climatic factors is complex. PH, EH and SD were significantly affected by RainB, DER and ED was significantly affected by TeffectB, EER and PDR was significantly affected by TminT, MDRV, EL, LGN, GNE and GW1000 was significantly affected by SolarT, EL was significantly affected by SolarA, ER was significantly affected by RainA, CD was significantly affected by TmaxB, BTL was significantly affected by RainT, KP was significantly affected by TmaxA, and GBD was significantly affected by EffectA (**Table 4**).

**Table 4:** Correlation analysis of plant agronomic traits, yield and meteorological factors.

	RainA	RainB	RainT	SolarA	SolarB	SolarT	TeffectA	TeffectB	TeffectT	TmaxA	TmaxB	TmaxT	TminA	TminB	TminT
PH	-0.06^*^	-0.24^***^	-0.21^***^	-0.06^**^	0.13^***^	0.05^*^	-0.08^**^	-0.13^***^	-0.16^***^	0.02	-0.01	0.01	-0.02	-0.20^***^	-0.14^***^
EH	0.11^***^	-0.19^***^	-0.06^**^	-0.13^***^	0.07^**^	-0.05^*^	-0.08^***^	-0.11^***^	-0.15^***^	-0.01	-0.02	-0.02	-0.02	-0.17^***^	-0.13^***^
SD	-0.05	-0.15^***^	-0.13^***^	-0.02	0.13^***^	0.08^*^	0.08^**^	0.09^**^	0.14^***^	0.07^*^	0.11^***^	0.14^***^	0.08^*^	0.08^**^	0.13^***^
DER	-0.05	0.01	-0.03	0.20^***^	0.05^*^	0.18^***^	0.01	-0.24^***^	-0.14^***^	-0.02	-0.16^***^	-0.13^***^	0.01	-0.17^***^	-0.09^**^
EER	0.13^***^	-0.08^*^	0.03	0.15^***^	0.11^***^	0.18^***^	0.27^***^	-0.03	0.23^***^	0.20^***^	-0.07^*^	0.13^***^	0.32^***^	-0.02	0.31^***^
LR	0.08	-0.02	0.04	0.03	0.01	0.03	0.01	-0.01	0.00	0.01	0.01	0.01	0.04	0.04	0.07
PDR	0.09^*^	0.06	0.11^**^	0.00	0.00	0.00	0.08^*^	0.11^**^	0.15^***^	0.09^*^	0.10^**^	0.15^***^	0.10^**^	0.13^***^	0.17^***^
MRDV	0.06	-0.01	0.03	0.17^***^	0.10^***^	0.20^***^	0.08^*^	-0.13^***^	-0.02	0.18^***^	-0.03	0.15^***^	0.13^***^	-0.08^*^	0.09^*^
EL	-0.17^***^	-0.05^*^	-0.15^***^	0.19^***^	0.08^**^	0.19^***^	0.06^*^	-0.13^***^	-0.03	-0.02	-0.05	-0.05^*^	-0.02	-0.15^***^	-0.10^***^
ER	0.14^***^	-0.13^***^	0.00	-0.04	0.02	-0.01	-0.01	-0.11^***^	-0.08^***^	-0.06^*^	-0.08^***^	-0.11^***^	0.01	-0.13^***^	-0.06^*^
LGN	-0.12^***^	-0.04	-0.11^***^	0.17^***^	0.13^***^	0.20^***^	0.02	-0.09^***^	-0.04	-0.05^*^	-0.01	-0.05^*^	-0.06^*^	-0.10^***^	-0.11^***^
GNE	-0.02	-0.08^**^	-0.07^***^	0.12^***^	0.09^***^	0.14^***^	0.04	-0.13^***^	-0.05^*^	-0.05	-0.05^*^	-0.08^***^	-0.01	-0.13^***^	-0.08^***^
ED	0.18^***^	-0.15^***^	0.01	-0.01	0.01	0.00	0.00	-0.24^***^	-0.15^***^	-0.01	-0.16^***^	-0.12^***^	0.10^***^	-0.20^***^	-0.02
CD	0.16^***^	0.04	0.13^***^	0.09^***^	-0.14^***^	-0.03	0.13^***^	-0.21^***^	-0.02	0.05^*^	-0.22^***^	-0.09^***^	0.18^***^	-0.11^***^	0.11^***^
BTL	-0.09^**^	-0.03	-0.08^***^	-0.28^***^	0.11^***^	-0.11^***^	-0.19^***^	0.27^***^	0.01	-0.13^***^	0.27^***^	0.05	-0.18^***^	0.23^***^	-0.05
KP	0.14^***^	0.07^**^	0.14^***^	0.07^**^	-0.03	0.02	0.16^***^	-0.02	0.13^***^	0.19^***^	-0.04	0.15^***^	0.16^***^	-0.08^**^	0.11^***^
GW1000	-0.08^**^	-0.15^***^	-0.16^***^	0.15^***^	0.12^***^	0.19^***^	0.16^***^	-0.07^**^	0.10^***^	0.15^***^	-0.04	0.11^***^	0.18^***^	-0.11^***^	0.11^***^
GBD	-0.07^**^	0.09^***^	0.02	0.14^***^	0.00	0.09^***^	0.19^***^	-0.05	0.13^***^	0.21^***^	-0.08^**^	0.15^***^	0.13^***^	-0.13^***^	0.05
Yield	-0.20^***^	-0.18^***^	-0.26^***^	0.17^***^	0.14^***^	0.23^***^	0.05^*^	-0.17^***^	-0.07^**^	0.02	-0.04	-0.01	0.02	-0.19^***^	-0.09^***^

PH, plant height; EH, ear height; SD, stem diameter; DER, Double ear rate; EER, empty ear rate; LR, lodging rate; PDR, Pour the discount rate; MRDV, maize rough dwarf virus; EL, ear length; ER, ear rows; LGN, line grain number; GNE, grain number per ear; ED, ear diameter; CD, cob diameter; BTL, bald tip long; KP, kernel percentage; GW1000, 1000 grains weight; GBD, grain bulk density; RainA, rainfall after silking; RainB, rainfall before silking; RainT, rainfall in total growth stage; SolarA, solar after silking; SolarB, solar before silking; SolarT, solar in total growth stage; TeffectA, effective accumulative temperature after silking; TeffectB, effective accumulative temperature before silking; TeffectT, effective accumulative temperature in total growth stage; TmaxA, Maximum accumulated temperature after silking; TmaxB, Maximum accumulated temperature before silking; TmaxT, Maximum accumulated temperature in total growth stage; TminA, Minimum cumulative temperature after silking; TminB, minimum cumulative temperature before silking; TminT, minimum cumulative temperature in total growth stage. *, significant correlation at the level of p < 0.05; **, significant correlation at the level of p < 0.01; ***, significant correlation at the level of p < 0.001.

### Contribution rate of agronomic traits and climatic factors to yield

The contribution rates of PH, DER, EER, PDR, MRDV, EL, GNE, ED, KP, GW1000 and GBD to the yield were 22.15%, 28.71%, -15.66%, -15.01%, -11.06%, 12.21%, 18.90%, 10.97%, 9.93%, 39.25%, 8.10%, respectively. The fitting equation could explain 54.44% of the yield variation (**Table 5**). In addition to the direct effect of agronomic traits on yield, there are also large indirect effects, such as the indirect path coefficient of EL through GNE is 0.119. The contribution rates of RainT, SolarB, SolarT, TeffectB, TmaxB, TmaxT, TminA and TminB to yield were -21.82%, -36.87%, 41.08%, -81.24%, 148.57%, -52.79%, 55.60%, -29.50%, respectively. And the fitting equation could account for 25.57% of yield variation (**Table 6**). In addition to the direct effect of climate factors on yield, there are also large indirect effects. For example, SolarB’s indirect path coefficient through TmaxB is 0.90.

**Table 5:** Contribution rate of plant agronomic traits to yield (Path analysis).

Factor			Regression coefficient		Standard regression coefficient		Partial correlation		t-values		p-values	
PH			0.0170		0.2215		0.2913		9.32		0.0000	
DER			0.1576		0.2871		0.3593		11.79		0.0000	
EER			-0.0571		-0.1566		-0.1920		5.99		0.0000	
PDR			-0.0321		-0.1501		-0.2081		6.51		0.0000	
MRDV			-0.0343		-0.1106		-0.1385		4.28		0.0000	
EL			0.1361		0.1221		0.1293		3.99		0.0001	
GNE			0.0048		0.1890		0.1971		6.15		0.0000	
ED			0.6478		0.1097		0.1284		3.96		0.0001	
KP			0.0544		0.0993		0.1342		4.15		0.0000	
GW1000			0.0181		0.3925		0.4292		14.55		0.0000	
GBD			-0.0041		-0.0810		-0.1108		3.41		0.0007	
Analysis of variance table												
Source of variation		Sum of squares			Degree of freedom		Mean square		F-values		p-values	
Regression		884.92			11		80.45		101.77		0.0000	
Residual error		740.70			937		0.79					
Total variation		1625.63			948							
Fitted equation		Yield=-10.69404561+0.017014083716^*^PH+0.15761213635^*^DER-0.05707024082^*^EER-0.03212117900^*^PDR-0.03426919004^*^MRDV+0.13610327896^*^EL+0.004764273130^*^GNE+ 0.6478093749^*^ED+ 0.05439949713^*^KP+0.018088229969^*^GW1000-0.004123812973^*^GBD										
R^2^		0.54										
Path analysis (Indirect coefficient)												
	PH		DER	EER	PDR	MRDV	EL	GNE	ED	KP	GW1000	GBD
PH			-0.027	0.011	-0.014	-0.008	0.006	0.015	0.018	0.024	0.055	-0.001
DER	-0.021			-0.034	0.001	-0.027	0.030	0.055	0.016	-0.002	0.010	-0.006
EER	-0.016		0.063		0.003	-0.050	0.001	0.005	0.018	-0.008	0.098	0.010
PDR	0.021		-0.002	0.004		0.001	0.015	0.001	-0.003	0.001	-0.060	0.010
MRDV	0.016		0.071	-0.070	0.001		0.016	0.030	0.020	-0.004	0.098	-0.004
EL	0.012		0.071	-0.002	-0.018	-0.015		0.119	0.042	-0.011	0.081	0.002
GNE	0.017		0.083	-0.004	-0.001	-0.018	0.077		0.041	0.005	0.026	-0.002
ED	0.035		0.041	-0.025	0.003	-0.020	0.047	0.071		-0.009	0.175	0.009
KP	0.053		-0.006	0.013	-0.002	0.005	-0.014	0.010	-0.010		-0.010	-0.018
GW1000	0.031		0.007	-0.039	0.023	-0.028	0.025	0.013	0.049	-0.002		-0.007
GBD	0.003		0.022	0.020	0.019	-0.006	-0.004	0.005	-0.012	0.022	0.035	

PH, plant height; EH, ear height; SD, stem diameter; DER, Double ear rate; EER, empty ear rate; LR, lodging rate; PDR, Pour the discount rate; MRDV, maize rough dwarf virus; EL, ear length; ER, ear rows; LGN, line grain number; GNE, grain number per ear; ED, ear diameter; CD, cob diameter; BTL, bald tip long; KP, kernel percentage; GW1000, 1000 grains weight; GBD, grain bulk density.

**Table 6:** Contribution rate of climatic factors to yield (Path analysis).

Factor		Regression coefficient		Standard regression coefficient			Partial correlation			t-values		p-values	
RainT		-0.0019		-0.2182			-0.22			9.43		0.0000	
SolarB		-0.0046		-0.3687			-0.17			7.11		0.0000	
SolarT		0.0038		0.4108			0.23			9.80		0.0000	
TeffectB		-0.0168		-0.8124			-0.26			11.56		0.0000	
TmaxB		0.0179		1.4857			0.33			14.91		0.0000	
TmaxT		-0.0044		-0.5279			-0.19			8.33		0.0000	
TminA		0.0062		0.5560			0.22			9.51		0.0000	
TminB		-0.0055		-0.2950			-0.13			5.66		0.0000	
Analysis of variance table													
Source of variation		Sum of squares		Degree of freedom			Mean square			F-values		p-values	
Regression		711.78		8			88.97			77.12		0.0000	
Residual error		2070.93		1795			1.15						
Total variation		2782.71		1803									
Fitted equation		Yield=7.02464818-0.0018935477315*RainT-0.004631368303*SolarB+ 0.003766535065*SolarT-0.016752902356*TeffectB+0.017885447143*TmaxB-0.004407768783*TmaxT+0.006228590234*TminA-0.005507587649*TminB											
R^2^		0.26											
Path analysis (Indirect coefficient)													
	RainT		SolarB		SolarT	TeffectB		TmaxB	TmaxT		TminA		TminB
RainT			0.12		-0.11	0.14		-0.39	0.06		0.12		0.02
SolarB	0.07				0.29	-0.38		0.90	-0.09		-0.16		-0.12
SolarT	0.06		-0.26			-0.11		0.27	-0.17		0.06		-0.03
TeffectB	0.04		-0.17		0.06			1.37	-0.25		-0.14		-0.26
TmaxB	0.06		-0.22		0.08	-0.75			-0.21		-0.23		-0.25
TmaxT	0.02		-0.06		0.13	-0.38		0.59			0.32		-0.10
TminA	-0.05		0.10		0.04	0.21		-0.62	-0.31				0.09
TminB	0.01		-0.15		0.05	-0.73		1.25	-0.18		-0.17		

RainA, rainfall after silking; RainB, rainfall before silking; RainT, rainfall in total growth stage; SolarA, solar after silking; SolarB, solar before silking; SolarT, solar in total growth stage; TeffectA, effective accumulative temperature after silking; TeffectB, effective accumulative temperature before silking; TeffectT, effective accumulative temperature in total growth stage; TmaxA, Maximum accumulated temperature after silking; TmaxB, Maximum accumulated temperature before silking; TmaxT, Maximum accumulated temperature in total growth stage; TminA, Minimum cumulative temperature after silking; TminB, minimum cumulative temperature before silking; TminT, minimum cumulative temperature in total growth stage.

## Discussion

The stability and high yield of varieties are important indexes for farmers’ selection. ZD958, as a variety developed in the 2000’s in the Huang-Huai-Hai region, still occupies a large market in this region (Ren et al., 2021), and is also the main research material for summer maize research in China (Hassan et al., 2020; Ren et al., 2021; Liu et al., 2021a; Liu et al., 2021b). The reason lies in its adaptability, high yield and stability to most climate zones in Huang-Huai-Hai region. In our study, we found that the average experimental yield in different regions of Shandong Province was 9.20 t ha^-1^, with the CV ranging from 4.55 to 20.82%, and the yield sustainability coefficient ranging from 0.57 to 0.9 (**Table 1**). The average CV of yield for all regions was 13.41%, which is far less than the CV for the stability of Argentina’s yield across regions reported by Di Matteo et al. (2016) in the last 45 years. It was also far less than the CV of summer maize yield (30%) in North China Plain reported by Zhao et al. (2018).

Based on the analysis of ZD958 agronomic traits, the relationships among different agronomic traits can be judged. There was a high positive correlation between PH and EH with a correlation coefficient of 0.69 (**Table 2**). Carpici and Celik (2010) identified a correlation coefficient of 0.847 between PH and EH, while Silva et al. (2016) reported a value of 0.919. The genes that influence both traits may be located at the same locus (Lu et al., 2020). The increase of PH and EH will lead to the increase of EL and ED, which will significantly increase ER, LGN, GNE and GW1000, so as to increase the yield. PH and EH were also significantly positively correlated with PDR, indicating that tall stems were prone to inverted folding (Sreckov et al., 2011). For maize yield, EER, PDR, and BTL all have significant negative effects on yield, so proper selection of PH is required. The increase of EER will lead to the increase of ED and CD, but the KP will decrease significantly, which will lead to the decrease of yield (**Figure 4**).

Climate change will have a significant effect on the agronomic traits and yield of crops (Hochman et al., 2017; Liu et al., 2021c). The change of climate conditions is the main reason for the variation of plant height, and the high temperature in the reproductive growth stage may promote the increase of plant height (Boomsma et al., 2010). Changes of climatic factors before and after anthesis are important factors affecting agronomic traits of maize. For example, excessive precipitation before anthesis will have a negative effect on plant height and ear height; changes of solar radiation have a significant positive correlation effect on yield; while effective accumulated temperature before anthesis has a significant negative correlation with yield. Compared with the maximum temperature, the increase of the minimum temperature has a more significant effect on the yield. Path analysis showed that RainT, SolarB, SolarT, TeffectB, TmaxB, TmaxT, TminA, TminB had a significant effect on yield and could account for 26% of yield variation (**Table 6**). Path analysis and correlation analysis are somewhat different, because path analysis considers the collinear relationship between factors and the indirect relationship between different factors (Tang, 2010).

The Huang-Huai-Hai Plain is a vast area with significant regional climate variation (Ren et al., 2021). According to our analysis of the characteristics of ZD958, it is necessary to have higher agronomic traits such as PH, EH, GNE and GW1000 in order to obtain higher yield. To maintain good yield stability, it is necessary to be insensitive to climate change, which requires maize to have stronger stress resistance, such as reduced EER, LR, PDR, and MRDV. The varieties with higher yield and better stability will be the varieties that farmers like.

## Conclusions

The results of 33 experimental stations in Shandong Province from 2005 to 2015 showed that it is difficult to obtain both super high yield and high stability of ZD958. The main agronomic traits that affected grain yield were PH, DER, EER, PDR, MRDV, EL, GNE, ED, KP, GW1000, GBD, and these factors accounted for 54% of the yield variation. Agronomic traits such as PH, EH, EL, ER, LGN, GNE, ED and GW1000 had significant positive correlation with yield. The variation of climate factors could explain 26% of the yield variation, which mainly affected the yield by affecting agronomic traits.

## Data availability statement

The original contributions presented in the study are included in the article/**Supplementary Material**. Further inquiries can be directed to the corresponding authors.

## Author contributions

PL, CL, ML, and HR contributed to conception and design of the study. PL, JZ and CL organized the database. ML performed the statistical analysis. HR wrote the first draft of the manuscript. ML, PL, JZ and CL wrote sections of the manuscript. All authors contributed to manuscript revision, read, and approved the submitted version.

## Funding

This study was supported by Key Research and Development Program of Shandong Province (LJNY202103), National Key Research and DevelopmentProgram of China (2016YFD0300106), Shandong Province Key Agricultural Project for Application Technology Innovation (SDAIT02-08), and Major scientific and technological innovation project in Shandong Province (2021CXGC010804-05).

## Conflict of interest

The authors declare that the research was conducted in the absence of any commercial or financial relationships that could be construed as a potential conflict of interest.

## Publisher’s note

All claims expressed in this article are solely those of the authors and do not necessarily represent those of their affiliated organizations, or those of the publisher, the editors and the reviewers. Any product that may be evaluated in this article, or claim that may be made by its manufacturer, is not guaranteed or endorsed by the publisher.

## References

[B1] Boomsma C. R. Santini J. B. West T. D. Brewer J. C. Mclntyre L. M. Vyn T. J. (2010). Maize grain yield responses to plant height variability resulting from crop rotation and tillage system in a long-term experiment. Soil Tillage Res. 106 (2), 227–240. doi: 10.1016/j.still.2009.12.006

[B2] Carpici E. B. Celik N. (2010). Determining possible relationships between yield and yield-related components in forage maize (Zea mays l.) using correlation and path analyses. Notulae Bot. Hort. Agrobot. Cluj-Napoca 38 (3), 280–285. doi: 10.15835/nbha3835431

[B3] Carter M. W. Poneleit C. G. (1973). Black layer maturity and filling period variation among inbred lines of corn (*Zea mays* l.). Crop Sci. 13 (4), 436–439. doi: 10.2135/cropsci1973.0011183X001300040014x

[B4] Chen F. Liu J. Liu Z. Chen Z. Mi G. (2021). Breeding for high-yield and nitrogen use efficiency in maize: lessons from comparison between Chinese and us cultivars. Adv. Agron. 166 (25), 251–275. doi: 10.1016/bs.agron.2020.10.005

[B5] Di Matteo J. A. Ferreyra J. M. Cerrudo A. A. Echarte L. Andrade F. H. (2016). Yield potential and yield stability of argentine maize hybrids over 45 years of breeding. Field Crops Res. 197, 107–116. doi: 10.1016/j.fcr.2016.07.023

[B6] Duvick D. N. (2005). The contribution of breeding to yield advances in maize (*Zea mays* l.). Adv. Agro. 86, 83–145. doi: 10.1016/S0065-2113(05)86002-X

[B7] Falcon C. M. Kaeppler S. M. Spalding E. P. Miller N. D. Haase N. AlKhalifah N.. (2020). Relative utility of agronomic, phenological, and morphological traits for assessing genotype-by-environment interaction in maize inbreds. Crop Sci. 60 (1), 62–81. doi: 10.1002/csc2.20035

[B8] Hassan M. U. Islam M. M. Wang R. Guo J. Luo H. Chen F. (2020). Glutamine application promotes nitrogen and biomass accumulation in the shoot of seedlings of the maize hybrid ZD958. Planta 251 (3), 1–15. doi: 10.1007/s00425-020-03363-9 32065312

[B9] Hochman Z. Gobbett D. L. Horan H. (2017). Climate trends account for stalled wheat yields in Australia since 1990. Global Change Biol. 23 (5), 2071–2081. doi: 10.1111/gcb.13604 28117534

[B10] Jia H. Li M. Liu L. Jian Y. Yang Z. Shen X.. (2020). A serine/threonine protein kinase encoding gene KERNEL NUMBER PER ROW6 regulates maize grain yield. Nat. Commun. 11 (1), 988. doi: 10.1038/s41467-020-14746-7 32080171 PMC7033126

[B11] Lee E. A. Tollenaar M. (2007). Physiological basis of successful breeding strategies for maize grain yield. Crop Sci. 47, S202–S215. doi: 10.2135/cropsci2007.04.0010IPBS

[B12] Li W. Ge F. Qiang Z. Lei Z. Zhang S. Chen L.. (2019). Maize zmrph1 encodes a microtubule-associated protein that controls plant and ear height. Plant Biotechnol. J. 18 (6), 1345–1347. doi: 10.1111/pbi.13292 31696605 PMC7206999

[B13] Liu Z. Hu C. Wang Y. Sha Y. Mi G. (2021a). Nitrogen allocation and remobilization contributing to low-nitrogen tolerance in stay-green maize. Field Crops Res. 263, 108078. doi: 10.1016/j.fcr.2021.108078

[B14] Liu G. Yang H. Xie R. Liu W. Guo X. Xue J.. (2021b). Genetic gains in maize yield and related traits for high-yielding cultivars released during 1980s to 2010s in China. Field Crops Res. 270, 108223. doi: 10.1016/j.fcr.2021.108223

[B15] Liu Y. Zhang J. Pan T. Ge Q. (2021c). Assessing the adaptability of maize phenology to climate change: The role of anthropogenic-management practices. J. Environ. Manage. 293 (5), 112874. doi: 10.1016/j.jenvman.2021.112874 34058454

[B16] Lu S. Li M. Zhang M. Lu M. Liu W. (2020). Genome-wide association study of plant and ear height in maize. Trop. Plant Biol. 13 (3), 262–273. doi: 10.1007/s12042-020-09258-z

[B17] Ren H. Han K. Liu Y. Zhao Y. Zhao B. (2021). Improving smallholder farmers’ maize yields and economic benefits under sustainable crop intensification in the north China plain. Sci. Total Environ. 763 (16), 143035. doi: 10.1016/j.scitotenv.2020.143035 33131864

[B18] Setimela P. S. Gasura E. Tarekegne A. T. (2017). Evaluation of grain yield and related agronomic traits of quality protein maize hybrids in southern Africa. Euphytica 213 (12), 289. doi: 10.1007/s10681-017-2082-2

[B19] Silva T. N. Moro G. V. Moro F. V. Santos D. M. M. D. Buzinaro R. (2016). Correlation and path analysis of agronomic and morphological traits in maize. Rev. Ciencia Agro. 47 (2), 351–357. doi: 10.5935/1806-6690.20160041

[B20] Simmons C. R. Lafitte H. R. Reimann K. S. Brugière N. Habben J. E. (2021). Successes and insights of an industry biotech program to enhance maize agronomic traits. Plant Sci. 307, 110899. doi: 10.1016/j.plantsci.2021.110899 33902858

[B21] Sreckov Z. Nastasic A. Bocanski J. Djalovic L. Vukosavljev M. Jockovic B. (2011). Correlation and path analysis of grain yield and morphological traits in test–cross populations of maize. Pakistan J. Bot. 43 (3), 1729–1731.

[B22] Tang Q. (2010). DPS data processing system (Beijing: Science Press).

[B23] Wang H. Zhang X. Hu F. Liu M. Zhao Y. Wang Y. (2019). Systematic identification and characterization of candidate genes for the regulation of plant height in maize. Euphytica 215 (2), 1. doi: 10.1007/s10681-019-2345-1

[B24] Zhao J. Yang X. Sun S. (2018). Constraints on maize yield and yield stability in the main cropping regions in china. Eur. J. Agron. 99, 106–115. doi: 10.1016/j.eja.2018.07.003

