# Supplementary material for: Effects of agronomic traits and climatic factors on yield and yield stability of summer maize (Zea mays L) in the Huang-Huai-Hai Plain in China
Source: Front Plant Sci. 2022 Nov 10;13:1050064. doi: 10.3389/fpls.2022.1050064 (PMC9707337; doi:10.3389/fpls.2022.1050064)
Supplement: Supplementary file 1 [file Table_1.docx]

Table S1 Basic meteorological conditions in summer maize growth period of the experimental station

| Site | Rainfall  (mm) | | Solar radiation  (MJ m^-2^) | | | Effective accumulative temperature (℃) | | Accumulated maximum temperature (℃) | | | Accumulated minimum temperature (℃) | | |
| --- | --- | --- | --- | --- | --- | --- | --- | --- | --- | --- | --- | --- | --- |
| Tengzhou | 619± | 144 | | 1536± | 121 | 1606± | 52 | | 3155± | 111 | | 2280± | 50 |
| Wenkou | 454± | 80 | | 1610± | 142 | 1472± | 83 | | 2986± | 141 | | 2080± | 90 |
| Juancheng | 372± | 84 | | 1511± | 149 | 1535± | 96 | | 3097± | 151 | | 2174± | 95 |
| Tancheng | 586± | 186 | | 1580± | 81 | 1534± | 69 | | 3074± | 97 | | 2241± | 74 |
| Pingyi | 567± | 151 | | 1649± | 140 | 1551± | 61 | | 3066± | 104 | | 2214± | 58 |
| Feicheng | 423± | 90 | | 1733± | 120 | 1557± | 53 | | 3216± | 95 | | 2193± | 59 |
| Lvge | 453± | 128 | | 1740± | 112 | 1442± | 37 | | 3012± | 90 | | 2083± | 52 |
| Yuncheng | 436± | 97 | | 1589± | 130 | 1557± | 77 | | 3136± | 123 | | 2234± | 77 |
| Jiayang | 381± | 132 | | 1612± | 88 | 1556± | 93 | | 3205± | 103 | | 2136± | 98 |
| Zaozhuang | 409± | 141 | | 1555± | 74 | 1610± | 95 | | 3153± | 133 | | 2254± | 109 |
| Jining | 469± | 138 | | 1671± | 106 | 1594± | 100 | | 3137± | 149 | | 2246± | 124 |
| Linqu | 391± | 131 | | 1806± | 119 | 1541± | 40 | | 3159± | 93 | | 2172± | 58 |
| Denghai | 451± | 166 | | 1863± | 73 | 1627± | 67 | | 3155± | 128 | | 2325± | 109 |
| Ningjin | 392± | 151 | | 1693± | 98 | 1505± | 46 | | 3098± | 89 | | 2103± | 54 |
| Changqing | 458± | 127 | | 1746± | 77 | 1592± | 43 | | 3218± | 55 | | 2255± | 58 |
| Haiyang | 442± | 177 | | 1689± | 178 | 1463± | 80 | | 2961± | 90 | | 2216± | 59 |
| Lizi | 423± | 149 | | 1610± | 113 | 1573± | 80 | | 3201± | 99 | | 2142± | 73 |
| Guanxian | 397± | 135 | | 1638± | 85 | 1524± | 52 | | 3132± | 90 | | 2142± | 72 |
| Jinxiang | 449± | 146 | | 1670± | 83 | 1595± | 73 | | 3139± | 80 | | 2240± | 79 |
| Zhangdian | 373± | 105 | | 1601± | 113 | 1608± | 60 | | 3171± | 95 | | 2223± | 75 |
| Nancun | 437± | 141 | | 1748± | 129 | 1455± | 49 | | 3007± | 124 | | 2108± | 66 |
| Ruzhong | 418± | 143 | | 1705± | 73 | 1526± | 48 | | 3107± | 49 | | 2138± | 42 |
| Huimin | 383± | 115 | | 1698± | 126 | 1679± | 41 | | 3315± | 84 | | 2313± | 54 |
| Jinhai | 414± | 141 | | 1875± | 79 | 1599± | 53 | | 3100± | 88 | | 2280± | 79 |
| Qingzhou | 380± | 161 | | 1706± | 131 | 1465± | 223 | | 2944± | 468 | | 2026± | 306 |
| Liaocheng | 371± | 120 | | 1560± | 81 | 1507± | 52 | | 3163± | 82 | | 2148± | 50 |
| Fengge | 416± | 116 | | 1749± | 55 | 1438± | 51 | | 3056± | 69 | | 2092± | 55 |
| Jiaozhou | 411± | 125 | | 1668± | 73 | 1482± | 74 | | 3018± | 67 | | 2206± | 66 |
| Weifang | 366± | 142 | | 1793± | 159 | 1490± | 35 | | 3046± | 79 | | 2071± | 42 |
| Dezhou | 449± | 196 | | 1774± | 102 | 1673± | 46 | | 3306± | 95 | | 2343± | 54 |
| Taian | 374± | 80 | | 1689± | 90 | 1561± | 45 | | 3133± | 57 | | 2177± | 44 |
| Juxian | 533± | 74 | | 1670± | 89 | 1498± | 51 | | 3117± | 21 | | 2173± | 31 |
| Liaolan | 414± | 135 | | 1725± | 152 | 1441± | 76 | | 2943± | 166 | | 2065± | 85 |
